# Kinetic study of Fe & Co perovskite catalyst in Fischer–Tropsch synthesis

**DOI:** 10.1038/s41598-024-59561-y

**Published:** 2024-04-22

**Authors:** Behnoosh Moshtari, Seyed Hasan Hashemabadi, Yahya Zamani

**Affiliations:** CFD Research Laboratory, School of Chemical Engineering, Iran University of Science and Technology, Narmak, Tehran, Iran

**Keywords:** Fischer–Tropsch synthesis, Perovskite catalyst, Kinetic model, Chemistry, Engineering

## Abstract

The investigation of the reaction's kinetics is one of the most crucial aspects of the design of a commercial process. The current research investigates the kinetics of Fischer–Tropsch synthesis using a perovskite catalyst. The LaFe_0.7_ Co_0.3_ O_3_ perovskite catalyst was prepared via the thermal sol–gel technique and characterized using BET, XRD, SEM, and H_2_-TPR techniques. According to operating conditions (e.g. H_2_/CO: 1–2, pressure: 10–20 barg, temperature: 240–300 °C, and GHSV: 3000 1/h), Fischer–Tropsch reaction kinetics (CO conversion) were carried out in a fixed-bed reactor. Using the framework of Langmuir–Hinshelwood–Hougen–Watson (LHHW) theories, 18 kinetic expressions for CO conversion were derived, and all were fitted to experimental data one by one to determine the optimum condition. The correlation was derived from experimental data and well-fitted using LHHW form (according to the enol mechanism, carbon monoxide and dissociated hydrogen atoms are adsorbed and reacted on the surface of the catalyst) −r_CO_ = k_p_b_CO_P_CO_(b_H2_P_H2_)^0.5^/(1 + b_CO_P_CO_ + (b_H2_P_H2_)^0.5^)^2^. Finally, the activation energy of the optimum kinetic model was determined with respect to the Arrhenius equation under various operating conditions. The activation energy of perovskite catalyst is about 106.25 kJ/mol at temperatures 240–300 °C, pressures 10–20 barg, and H_2_/CO ratios 1–2, which is lower than other types of catalyst. Therefore, the catalyst was activated at a high temperature and demonstrated stable performance without any temperature runaway and coking issues.

## Introduction

Fischer–Tropsch synthesis (FTS) is a series of polymeric reactions for converting syngas (a mixture of CO and H_2_) to liquid hydrocarbons^1–3^. The high growth of the world's population and the advancement of technology have led to an increase in the demand for liquid fuels; consequently, this process is regarded as a viable alternative for producing valuable fuels^4–6^ Hydrocarbons with FTS are considered clean fuels due to their low sulfur content^7^. FT process is regarded as a catalytic process. Metals in the 8–10 group are the most effective catalysts for Fischer–Tropsch synthesis, and among them, Iron and cobalt have demonstrated superior performance compared to other metals. Due to the exothermic nature of the reactions in the FT process, catalyst efficiency reduces accordingly. Therefore, the yield of desirable products decreases due to the resistance of the catalyst to a sudden increase in temperature; thus the behavior of the catalyst at the beginning of the reaction is crucial. Perovskite catalysts have been popular in recent years due to their high thermal stability and diverse performance^7,8^. The general structure of the perovskite catalysts is ABO_3_, in which A and B are metal groups and A is a larger cation than B^8–10^. Furthermore, investigating the kinetic is one of the most important topics for designing commercial processes. Therefore, many researchers are interested in developing a kinetic model for the FT process with a wide range of operating conditions^11,12^. There have been numerous attempts to define the FT process's kinetics, and there are many kinetic models. All the developing models can be classified into 4 categories: Power Low (PL), Eley-Raidal (ER), Langmuir Hinshelwood Hugon Watson (LHHW), and Termolecular (TM)^13–16^. Anderso, Dry and Yates suggested the first kinetic equations for Fischer Tropsch synthesis, and it was widely used by other researchers^17–19^. Boots et al. studied the kinetic equations for Fe and Co catalysts in various operating conditions. They proved that the empirical results demonstrate a significant deviation from model equations. They reported new kinetic equations (Table 1)^20,21^. In 2010 Ojeda et al.^22^ investigated the kinetics of Fischer-Trosch synthesis using iron and cobalt catalysts. They stated that the same mechanism could explain the kinetic model of Iron and cobalt, and finally, they developed a single equation for both catalysts (Table 1). Although the actual partial pressure of hydrogen is zero, their predicted model is not zero; therefore, it is not acceptable^22^. Nikbakht et al.^23^ reported the catalyst performance along with the kinetic of hydrocarbon-formation reactions for a Fe-Co-Ce catalyst on zeolite support in a fixed bed reactor. They developed the best-fitted model and obtained the kinetic parameters for CO consumption with/without the water term. Einbeigi et al. investigated the kinetics and mechanisms of Fischer–Tropsch synthesis over a %10Fe/%10Co/%80 γ-Al_2_O_3_ nanocatalyst prepared by the impregnation method in a fixed bed micro-reactor^24^. In 2023, Yahyazadeh et al. evaluated the kinetics of the FT reaction with a KMo bimetallic-promoted Fe catalyst supported on carbon nanotubes (CNTs)^25^. Although there are many studies on the kinetic models of Fischer–Tropsch synthesis using Fe or Co as an active site with various supporters (e.g. γ-alumina or TiO_2_), few studies have been carried out on the kinetic models of perovskite catalysts. Due to high thermal stability and low deactivation rate, perovskite catalysts have been noticed recently. Therefore, the current research focused on the kinetic and mechanism of Fischer–Tropsch based on perovskite catalyst; that is the LaFe_0.7_ Co_0.3_ O_3_ perovskite catalyst is prepared, characterized and tested in a fixed bed reactor.

**Table 1:** Most cited Fischer–Tropsch reaction rate equations.

Equation	catalyst	Type	Ref
$A{P}_{{H}_{2}}^{0.5}{P}_{CO}^{-0.2}$	Co/Al_2_O_3_	PL	^13^
$A\frac{{P}_{{H}_{2}}^{0.5}{P}_{CO}^{-0.25}}{1-{k}_{1}\left(\frac{{P}_{{H}_{2}O}}{{P}_{{H}_{2}}}\right)}$	Co/SiO_2_	ER	^14^
$A\frac{{P}_{{H}_{2}}{P}_{CO}^{0.5}}{{(1+{k}_{1}{P}_{CO}^{0.5})}^{3}}$	Co/Al_2_O_3_	TM	^15^
$\frac{k{P}_{CO}{P}_{{H}_{2}}}{{\left(1+a{P}_{{H}_{2}}^{0.5}+b{P}_{CO}{P}_{{H}_{2}}^{0.5}\right)}^{2}}$	Co/MnO_4_	LHHW	^16^
$\frac{A{P}_{{H}_{2}}{P}_{CO}+B{P}_{CO}}{{(1+{k}_{1}{P}_{CO})}^{2}}$	Fe,Co/K,Cu	LHHW	^22^

## Material and methods

### Materials

All materials were used as received: Iron (III) nitrate (Fe(NO_3_)_3_.9H_2_O, Merck, 98%), Cobalt (II) nitrate (Co(NO_3_)_3_.6H_2_O, Merck, 99%), Lanthanum(III) nitrate (La(NO_3_)_3_.6H_2_O, Merck, 99%), Glycine (NH_2_CH_2_COOH, Merck, 99%). The deionized water was applied for catalyst synthesis.

### Methods

#### Catalyst preparation

There are various methods for preparing perovskite oxide such as hydrothermal, microwave, precipitation, sol–gel, etc.^26^. The La Fe_0.7_ Co_0.3_ O_3_ perovskite catalyst was prepared using the thermal sol–gel method. The catalyst was synthesized at room temperature by an aqueous solution of La(NO_3_)0.6H_2_O, Co(NO_3_)0.6H_2_O, and Fe(NO_3_)0.9H_2_O, then glycine solution was added drop wise to form the gel^27,28^. The prepared gel was dried at 180 °C for 4 h in the oven, and then calcinated in a furnace at 550 °C (5 °C/min) for 5 h. The sample was pressed into pellets, crushed, and sieved to obtain particles in size between the 40–60 ASTM mesh.

#### Catalyst characterization

The XRD measurement was carried out using the Philips PW1729 system. The diffraction pattern was obtained using a CuKα lamp with wavelength λ = 1.542°A in the angle range 2θ = 1 to 2θ = 80 and a step size of 0.06. The size of the particle crystals is calculated based on the XRD information and using the Scherer equation.

Brunner Emmett Teller (BET) surface area, pore volume and mean pore diameter of the catalyst were determined using an ASAP 3020 instrument of Micrometrics. The SEM images were recorded using MIRA3 Tescan. Temperature Programmed Reduction (TPR) was carried out on 0.011 g of catalyst heated from 28 to 750 °C (20 °C/min) under 5% H_2_ in Argon (total gas flow: 50 Ncc/min) with Chembet 3000 system. A CM120 microscope manufactured by Philips did the Transmission Electron Microscopy (TEM) test on the catalysts.

#### Catalyst activity measurement

**Figure 1:**
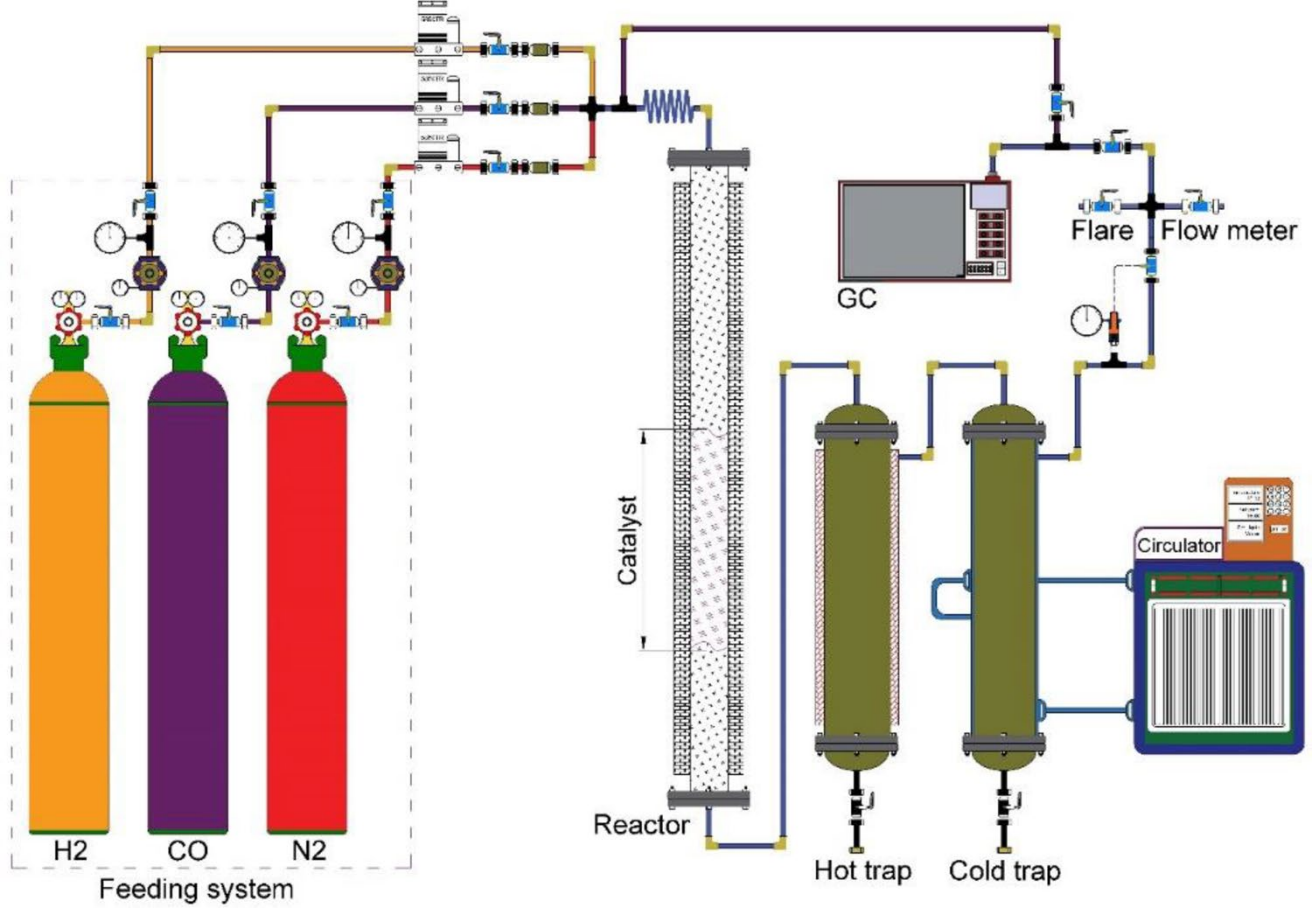
Schematics of the Fischer–Tropsch Set-up.

The kinetic tests were performed in a fixed bed reactor using LaFe_0.7_ Co_0.3_ O_3_ catalyst for Fischer Tropsch synthesis. The reactor was made of stainless steel with an 8 mm diameter and 700 mm height. The amount of 1 g catalyst is loaded into one-third of the reactor. The set-up consisted of 3 units: a feeding unit, a reaction unit, and a product separation and analysis unit. Figure 1 shows the schematic of the FT set-up.

The catalyst performance was tested under pressure and temperature ranges of 10–20 bar and 240–300 °C, respectively. The feed consisted of carbon monoxide and hydrogen with a percentage of 50% and 50%, respectively. The experimental results are reported in Table 3. The reactor temperature is controlled by a furnace equipped with a temperature controller located around the reactor to maintain a uniform temperature. To prevent choking of the reactor’s outlet in case of the formation of heavy hydrocarbons, the reactor system is equipped with two hot and cold traps to collect light and heavy liquid products. The liquid products are discharged from the traps and gas products are sent to the GC (Agilent 7890) by a stainless steel tube. The feed has three mass flow controllers (MFCs): hydrogen, carbon monoxide, and nitrogen gases. The feed was preheated prior to entering the reactor. The reactor's pressure is adjusted using a pressure valve (Groove) installed before the outlet (Fig. 1).

After loading the catalyst in the reactor, to reduce the catalyst, the mixture of hydrogen and nitrogen gases with a ratio of 1 to 10 (H_2_ / N_2_ = 1/10) under atmospheric pressure at 450 °C for 24 h passes throughout the catalyst bed. However, developing kinetic equations requires a series of experiments in specific operating conditions. The intrinsic rate equations were estimated by comparing the theoretical and experimental rates. To determine the kinetic equation in a fixed bed reactor, several factors should be considered as follows^19,29,30^:

- Catalyst activity should not be reduced.
- The temperature of the reactor should be constant (Due to the exothermic nature of the Fischer reaction, the operating conditions of the reaction should be considered in such a way that the CO conversion is low.)
- No mass transfer limitation is implied in the calculations.

However, to determine the kinetic equation under laboratory conditions, the velocity must be assumed to be uniform along the length of the reactor. Regarding the above assumption, the operating conditions should be implemented in such a way that the CO conversion becomes less than 15%. In these situations, the reaction rate through the reactor is constant and the following equation can be used:

1 $$\frac{W}{{F}_{CO}}={\int }_{{x}_{in}}^{{x}_{out}}\frac{dx}{-{r}_{CO}}=\frac{{X}_{out}-{X}_{in}}{{-r}_{CO}}$$

The average rate is:

2 $${-r}_{CO}=\frac{{x}_{CO}{F}_{CO}}{W} or {-r}_{CO}={r}_{CO (in)}-{r}_{CO (out)}$$

In order to simplify the kinetics equations, the mass transfer resistance was relinquished from calculations.

According to the ideal gas low the partial pressure of each component calculated as blow:

3 $${P}_{co}={X}_{co}*{P}_{total}$$

4 $${P}_{H2}={X}_{H2}*{P}_{total}$$

## Result and discussion

### Catalyst characterization results

**Figure 2:**
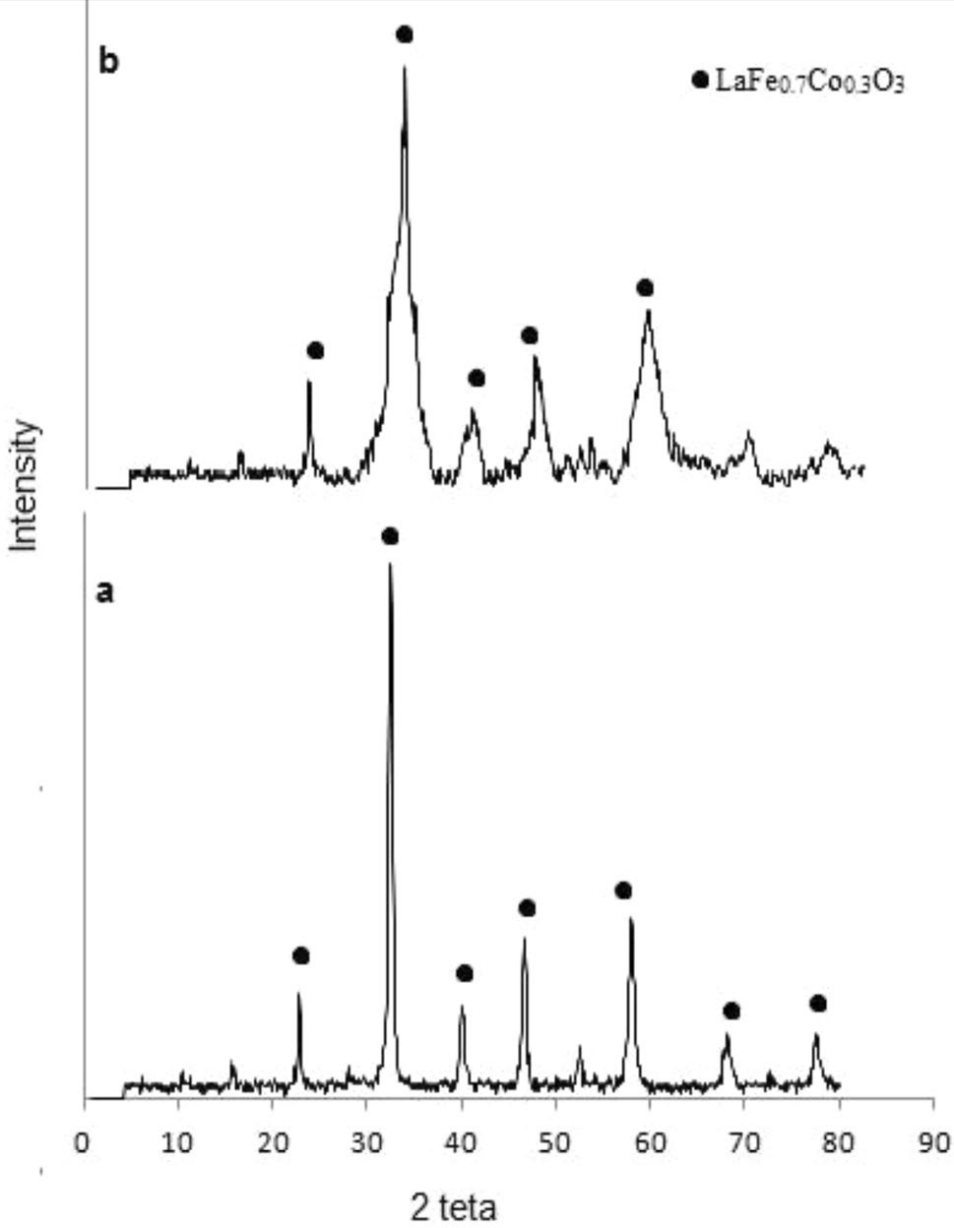
XRD Pattern (**a**) fresh catalyst (**b**) used catalyst.

The X-ray diffraction (XRD) of the prepared LaFe_0.7_Co_0.3_O_3_ is presented in Fig. 2a. The catalyst was calcinated at 550 °C and XRD determined the crystal structure. According to Fig. 2 the diffraction peaks of the perovskite structure in the range of 2θ = 30–40 are sharpened, and it is proved that the perovskite structure was formed. The used catalyst was characterized using XRD technique to identify the changes in its structure (Fig. 2b). The size of the LaFe_0.7_Co_0.3_O_3_ crystals was calculated according to X-ray diffraction data with Scherer's equation^31^:

5 $$d=\frac{K\lambda }{\beta\,cos\theta }$$

Scherer modified another correlation to calculate the size of crystals^31^:

6 $$ln\beta =ln\frac{k\lambda }{L}+ln\frac{1}{cos\theta }$$

**Figure 3:**
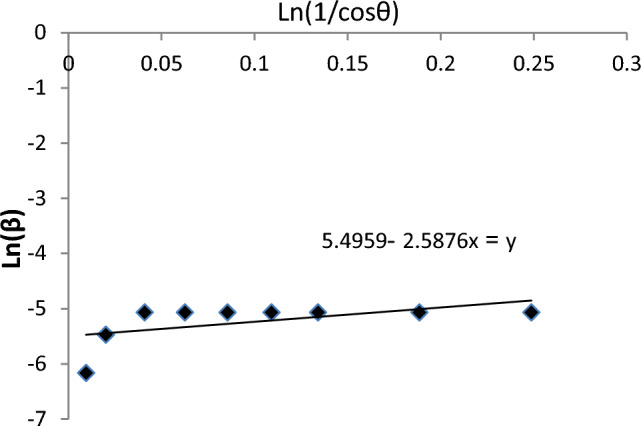
Lnβ verses Ln(1/cosθ) based on XRD results.

According to Scherer's equation, the average particle size was 30.69. The Ln(β) vs. Ln(1/cosθ) line graph was depicted for the LaFe_0.7_Co_0.3_O_3_ crystals using the modified Scherer correlation (Fig. 3). According to the correlation, the predicted size is 33.81. The size difference between the two predicted models is less than 4. Hence, the error percentage is insignificant, so both correlations are acceptable, but the modified Scherer is more compatible than others.

The BET test was performed to determine the specific surface area of the catalyst after calcination at 550 °C (Table 2). Whereas, the surface area of the perovskite catalysts is low, but its strong structure makes it valuable.

**Table 2:** BET results.

	Surface area (m^2^/gr)	Pore volume (cm^3^/gr)	Pore size (nm)
LaFe_0.7_Co_0.3_O_3_ (Fresh)	23.7	0.0959	24.2
LaFe_0.7_Co_0.3_O_3_ (Used)	10.3	0.0625	27.6

**Figure 4:**
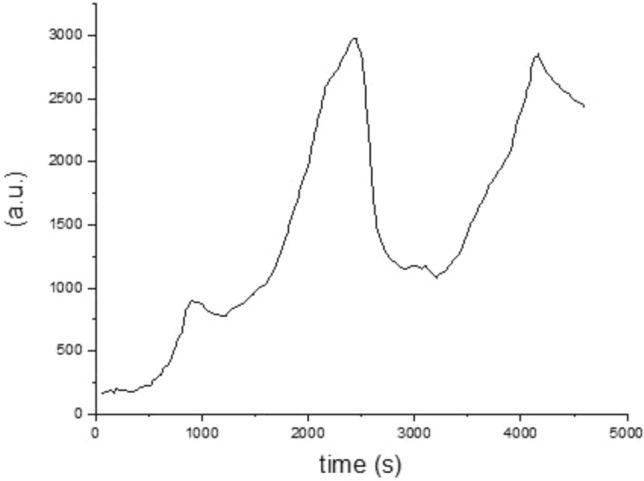
TPR profile of the perovskite catalyst.

Figure 4 represents the hydrogen consumption of the catalyst after the calcination process. As reported in Fig. 4 there are two reduction peaks between 2000 and 4000 s. The current result is inline with previous studies^11,32–34^. The first reduction trend appears at low temperatures, roughly 280 °C in the 2500 s, while the second trend appears at temperatures between 380 and 430 °C in the 4200 s. Two peaks in the TPR profile reflect the two reducible cations in the B site (Fe and Co)^11^.

**Figure 5:**
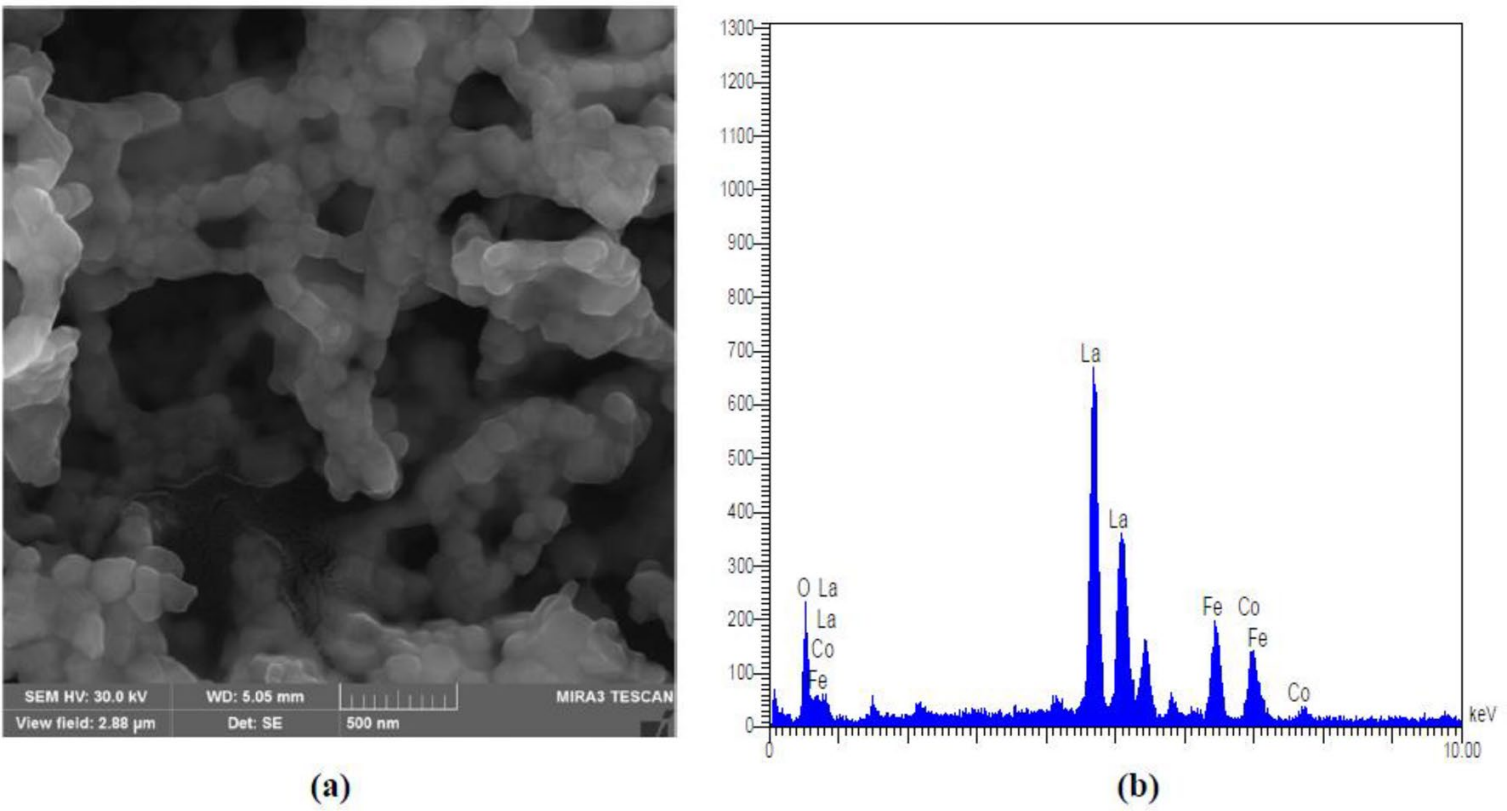
(**a**) SEM micrographs and (**b**) EDX Spectrum.

SEM images in Fig. 5a show that porous particles were formed and the EDXS proved that the actual composition of the catalyst is LaFe_0.67_Co_0.32_O_3_ (Fig. 5b).

**Figure 6:**
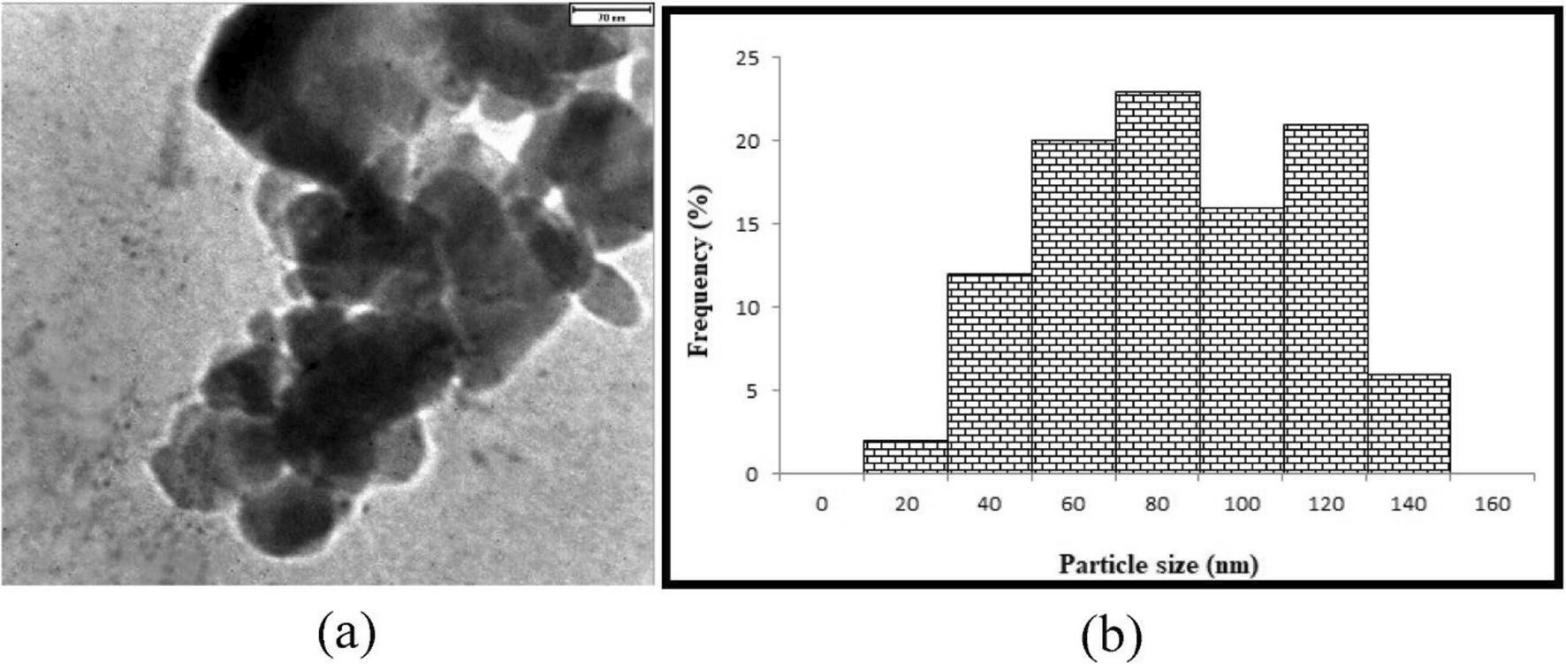
TEM Pattern (**a**) Schematic of catalyst, and (**b**) particle size distribution.

TEM observation of samples shows good distributions of grains of catalysts (Fig. 6). The results show that the particle size is between 20 and 140 nm, and the asymmetric shape of the grains proves that the XRD pattern is accurate.

### Kinetic model

The main goal of the current research is to develop an appropriate kinetic model to predict the behavior of the perovskite catalyst (LaFe_0.7_Co_0.3_O_3_) used for the Fischer–Tropsch process. The most crucial aspect of the FT process is elucidating the mechanism of the reaction. Moreover, the mechanisms were determined using various adsorption possibilities of CO and H_2_ molecules on the catalyst’s surface. There are four mechanisms for FT synthesis, such as carbide, enolic, alkyl, and alkenyl^23,35,36^. However, the main difference between the mechanisms is the monomer formation; the monomer formation stage was used to estimate the reaction rate.

**Figure 7:**
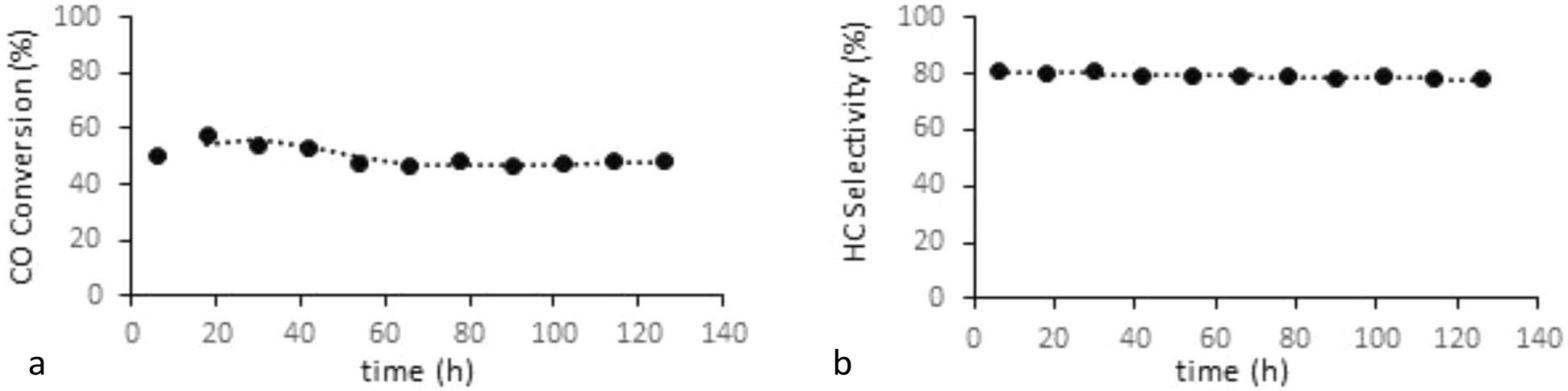
Activity diagram during the time (**a**) CO convention (**b**) Hydrocarbon Selectivity.

**Figure 8:**
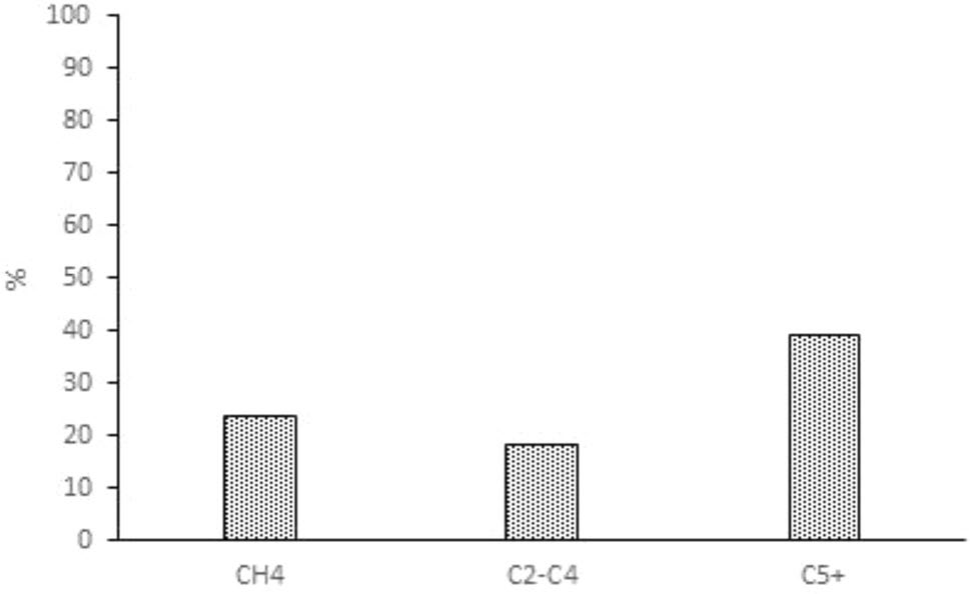
Product selectivity of LaFeCoO catalyst at a temperature of 553 K and pressure of 20 bar.

The activity of LaFe_0.7_CO_0.3_O_3_ perovskite catalyst was studied (Fig. 7) and the results show that after 50 h, the catalyst activity was stable. Additionally, in order to mitigate the impact of deactivation, new catalysts were loaded into each experiment. Therefore the kinetic data is reliable. The product selectivity at the temperature of 553 K and pressure of 20 bar within the catalyst activity range is reported (Fig. 8). The results show that the selectivity of C_5_^+^ is in good agreement with previous studies^8^. Table 3 shows the elementary reaction sets for FT synthesis. The reaction rate expressions for the FTS based on elementary reactions are shown in Table 4. In the current study, to derive all reaction rates, represented in Table 5, the Langmuir–Hinshelwood–Hougen–Watson (LHHW) approach was applied accordingly. In addition, using polymath software, a suitable kinetic model was developed by fitting whole reaction rates against empirical data, and finally, the best kinetic model was chosen based on R^2^, R_msd_, and MARR.

**Table 3:** Experimental condition and results for kinetic tests.

No	T(K)	H_2_/CO	P_CO_ (bar)	P_H2_ (bar)	X_CO_ (%)	X_H2_ (%)	− r_CO_ *10^−3^ (mol/min. gr_cat_)
1	513.15	1.0	10.0	10.0	3.36	2.89	1.9699
2	513.15	2.0	3.3	6.7	11.15	20.74	2.1750
3	553.15	2.0	3.3	6.7	11.59	21.09	2.0976
4	553.15	1.0	5.0	5.0	7.02	6.18	1.9069
5	533.15	1.5	6.0	9.0	6.53	8.36	2.2094
6	553.15	2.0	6.7	13.3	6.69	12.71	2.4250
7	513.15	2.0	6.7	13.3	6.16	11.58	2.4085
8	533.15	1.0	10.0	10.0	3.59	3.12	2.0234
9	533.15	2.0	3.3	6.7	11.48	21.70	2.1557
10	573.15	2.0	3.3	6.7	11.97	22.74	2.0903
11	573.15	1.0	5.0	5.0	7.27	6.54	1.9061
12	553.15	1.5	6.0	9.0	6.76	9.15	2.2047
13	573.15	2.0	6.7	13.3	7.00	13.30	2.4495
14	533.15	2.0	6.7	13.3	6.47	12.23	2.4357
15	573.15	1.0	10.0	10.0	3.69	3.28	1.9376
16	553.15	1.0	7.5	7.5	4.84	4.16	1.9730
17	513.15	2.0	3.3	6.7	10.75	20.10	2.0981
18	553.15	1.0	5.0	5.0	6.99	6.22	1.9006
19	553.15	2.0	3.3	6.7	11.55	21.13	2.0900
20	533.15	1.5	6.0	9.0	6.47	8.35	2.1890
21	553.15	1.0	10.0	10.0	3.61	3.32	1.9621
22	513.15	2.0	3.3	6.7	11.06	20.68	2.1581
23	553.15	1.0	5.0	5.0	6.88	6.19	1.8706
24	553.15	2.0	3.3	6.7	11.55	21.13	2.0900
25	533.15	1.5	6.0	9.0	6.53	8.42	2.2090
26	533.15	1.5	6.0	9.0	6.64	8.76	2.2467
27	533.15	1.5	6.0	9.0	6.60	8.58	2.2325
28	533.15	1.5	6.0	9.0	6.53	8.55	2.2111
29	513.15	1.0	5.0	5.0	6.48	5.51	1.8980
30	503.15	2.0	6.7	13.3	5.86	10.84	2.3373
31	513.15	2.0	6.7	13.3	6.23	11.71	2.4355
32	513.15	1.0	10.0	10.0	3.36	2.89	1.9695
33	533.15	1.5	6.0	9.0	6.54	8.50	2.2131

**Table 4:** Elementary reaction sets for Fischer–Tropsch synthesis.

Model	No	Reaction scheme	Model	No	Reaction scheme
FT-I	1	$CO+*\leftrightarrow {CO}^{*}$	FT-X	1	$CO+*\leftrightarrow {CO}^{*}$
	2	${CO}^{*}+{H}_{2}\leftrightarrow {CHOH}^{*}$		2	${CO}^{*}+*\leftrightarrow {C}^{*}+{O}^{*}$
	3	${CHOH}^{*}+{H}_{2}\leftrightarrow {CH}^{*}+{H}_{2}O$		3	${C}^{*}+{O}^{*}+{2H}_{2}\leftrightarrow {CH}_{3}{OH}^{*}+*$
FT-II	1	${H}_{2}+*\leftrightarrow {H}_{2}^{*}$		4	${CH}_{3}{OH}^{*}\leftrightarrow {CH}_{2}^{*}+{H}_{2}O$
	2	$CO+{H}_{2}^{*}\leftrightarrow {CH}_{3}{OH}^{*}$	FT-XI	1	$CO+*\leftrightarrow {CO}^{*}$
	3	${CH}_{3}{OH}^{*}\leftrightarrow {CH}_{2}^{*}+{H}_{2}O$		2	${CO}^{*}+*\leftrightarrow {C}^{*}+{O}^{*}$
FT-III	1	${H}_{2}+*\leftrightarrow {H}_{2}^{*}$		3	${C}^{*}+{O}^{*}+{H}_{2}\leftrightarrow {{CH}_{2}}^{*}+{O}^{*}$
	2	$CO+{H}_{2}^{*}\leftrightarrow {CHOH}^{*}$		4	${CH}_{2}^{*}+{O}^{*}+{H}_{2}\leftrightarrow {CH}_{3}{OH}^{*}+*$
	3	${CHOH}^{*}+{H}_{2}\leftrightarrow {CH}_{3}{OH}^{*}$		5	${CH}_{3}{OH}^{*}\leftrightarrow {CH}_{2}^{*}{+H}_{2}O$
	4	${CH}_{3}{OH}^{*}\leftrightarrow {CH}_{2}^{*}{+H}_{2}O$	FT-XII	1	$CO+{2}^{*}\leftrightarrow {C}^{*}{+O}^{*}$
FT-IV	1	${H}_{2}+2*\leftrightarrow {2H}^{*}$		2	${H}_{2}+{2}^{*}\leftrightarrow {2H}^{*}$
	2	$CO+{4H}^{*}\leftrightarrow {CH}_{3}{OH}^{*}$		3	${C}^{*}+{O}^{*}+{4H}^{*}\leftrightarrow {CH}_{3}{OH}^{*}+{2}^{*}$
	3	${CH}_{3}{OH}^{*}\leftrightarrow {CH}_{2}^{*}+{H}_{2}O$		4	${CH}_{3}{OH}^{*}\leftrightarrow {CH}_{2}^{*}{+H}_{2}O$
FT-V	1	${H}_{2}+{2}^{*}\leftrightarrow 2H$	FT-XIII	1	$CO+{2}^{*}\leftrightarrow {C}^{*}{+O}^{*}$
	2	$CO+{2H}^{*}\leftrightarrow {CHOH}^{*}$		2	${H}_{2}+{2}^{*}\leftrightarrow {2H}^{*}$
	3	${CHOH}^{*}+{H}_{2}\leftrightarrow {CH}_{3}{OH}^{*}$		3	${C}^{*}+{O}^{*}+{2H}^{*}\leftrightarrow {{CH}_{2}}^{*}+{O}^{*}+*$
	4	${CH}_{3}{OH}^{*}\leftrightarrow {CH}_{2}^{*}+{H}_{2}O$		4	${CH}_{2}^{*}+{O}^{*}+{2H}^{*}\leftrightarrow {CH}_{3}{OH}^{*}+{2}^{*}$
FT-VI	1	${H}_{2}+{2}^{*}\leftrightarrow {2H}^{*}$		5	${CH}_{3}{OH}^{*}\leftrightarrow {CH}_{2}^{*}{+H}_{2}O$
		$CO+{2H}^{*}\leftrightarrow {COH}^{*}$	FT-XIV	1	$CO+{2}^{*}\leftrightarrow {C}^{*}{+O}^{*}$
		${CHO}^{*}+{H}^{*}\leftrightarrow {CH}_{2}{OH}^{*}+*$		2	${H}_{2}+*\leftrightarrow {H}_{2}^{*}$
		${CH}_{2}{OH}^{*}+{H}^{*}\leftrightarrow {CH}_{3}{OH}^{*}+*$		3	${C}^{*}+{O}^{*}+{H}_{2}^{*}\leftrightarrow {CH}_{2}^{*}+{O}^{*}+*$
		${CH}_{3}{OH}^{*}\leftrightarrow {CH}_{2}^{*}{+H}_{2}O$		4	${CH}_{2}^{*}+{O}^{*}+{H}_{2}^{*}\leftrightarrow {CH}_{3}{OH}^{*}+{2}^{*}$
FT-VII	1	$CO+*\leftrightarrow {CO}^{*}$		5	${CH}_{3}{OH}^{*}\leftrightarrow {CH}_{2}^{*}{+H}_{2}O$
	2	${H}_{2}+{2}^{*}\leftrightarrow {2H}^{*}$	FT-XV	1	$CO+{2}^{*}\leftrightarrow {C}^{*}+{O}^{*}$
	3	$CO+{H}^{*}\leftrightarrow {CHO}^{*}+*$		2	${C}^{*}+{H}_{2}\leftrightarrow {CH}_{2}^{*}+*$
	4	${CHO}^{*}+{H}^{*}\leftrightarrow {CHOH}^{*}+*$	FT-XVI	1	$CO+{2}^{*}\leftrightarrow {C}^{*}{+O}^{*}$
	5	${CHOH}^{*}+{H}^{*}\leftrightarrow {CH}_{2}{OH}^{*}+*$		2	${H}_{2}+{2}^{*}\leftrightarrow {2H}^{*}$
	6	${CH}_{2}{OH}^{8}+{H}^{*}\leftrightarrow {CH}_{3}{OH}^{*}+*$		3	${C}^{*}+{H}^{*}\leftrightarrow {CH}^{*}+*$
	7	${CH}_{3}{OH}^{*}\leftrightarrow {CH}_{2}^{*}+{H}_{2}O$		4	${CH}^{*}+{H}^{*}\leftrightarrow {CH}_{2}^{*}$
FT-VIII	1	$CO+*\leftrightarrow {CO}^{*}$	FT-XVII	1	$CO+{2}^{*}\leftrightarrow {C}^{*}{+O}^{*}$
	2	${H}_{2}+{2}^{*}\leftrightarrow {2H}^{*}$		2	${H}_{2}+*\leftrightarrow {H}_{2}^{*}$
	3	${CO}^{*}+{4H}^{*}\leftrightarrow {CH}_{3}{OH}^{*}+*$		3	${C}^{*}+{H}_{2}^{*}\leftrightarrow {CH}_{2}^{*}+*$
	4	${CH}_{3}{OH}^{*}\leftrightarrow {CH}_{2}^{*}+{H}_{2}O$	FT-XVIII	1	$CO+{2}^{*}\leftrightarrow {C}^{*}{+O}^{*}$
FT-IX	1	$CO+*\leftrightarrow {CO}^{*}$		2	${H}_{2}+{2}^{*}\leftrightarrow {2H}^{*}$
	2	${H}_{2}+{2}^{*}\leftrightarrow {2H}^{*}$		3	${C}^{*}+{2H}^{*}\leftrightarrow {{CH}_{2}}^{*}+*$
	3	${CO}^{*}+{H}_{2}\leftrightarrow {CH}_{2}{O}^{*}+*$			
	4	${CH}_{2}{O}^{*}+{H}_{2}^{*}\leftrightarrow {CH}_{3}{OH}^{*}+*$			
	5	${CH}_{3}{OH}^{*}\leftrightarrow {CH}_{2}^{*}+{H}_{2}O$

**Table 5:** Reaction rates for Fischer–Tropsch synthesis.

Model	Rate equation	Model	Rate equation
FT-I (1)	${-r}_{CO}=\frac{k.{b}_{CO}.{P}_{CO}.{P}_{{H}_{2}}}{1+{b}_{CO}.{P}_{CO}}$	FT-X (1)	${-r}_{CO}=\frac{{k}_{CO}.{b}_{CO}.{P}_{CO}}{{\left(1+2{\left({b}_{CO}.{P}_{CO}\right)}^\frac{1}{2}\right)}^{2}}$
FT-I (2)	${-r}_{CO}=\frac{k.{b}_{CO}.{P}_{CO}}{1+{b}_{CO}.{P}_{CO}}$	FT-X (2)	${-r}_{CO}=\frac{{k}_{CO}.{b}_{CO}.{P}_{CO}.{{(P}_{{H}_{2}})}^{2}}{{\left(1+2{\left({b}_{CO}.{P}_{CO}\right)}^\frac{1}{2}\right)}^{2}}$
FT-II (1)	${-r}_{CO}=\frac{{k}_{{H}_{2}}.{b}_{{H}_{2}}.{P}_{{H}_{2}}}{1+{b}_{{H}_{2}}.{P}_{{H}_{2}}}$	FT-XI	${-r}_{CO}=\frac{{k}_{CO}.{b}_{CO}.{P}_{CO}.{P}_{{H}_{2}}}{{\left(1+2{\left({b}_{CO}.{P}_{CO}\right)}^\frac{1}{2}\right)}^{2}}$
FT-II (2)	${-r}_{CO}=\frac{{k}_{{H}_{2}}.{{{P}_{CO}.(b}_{{H}_{2}}.{P}_{{H}_{2}})}^{2}}{{(1+{b}_{{H}_{2}}.{P}_{{H}_{2}})}^{2}}$	FT-XII (1)	${-r}_{CO}=\frac{{k}_{CO}.{b}_{CO}.{P}_{CO}}{{\left(1+2{\left({b}_{CO}.{P}_{CO}\right)}^\frac{1}{2}.{\left({b}_{{H}_{2}}.{P}_{{H}_{2}}\right)}^\frac{1}{2} \right)}^{2}}$
FT-III	${-r}_{CO}=\frac{{k.P}_{CO}.{b}_{{H}_{2}}.{P}_{{H}_{2}}}{1+{b}_{{H}_{2}}.{P}_{{H}_{2}}}$	FT-XII (2)	${-r}_{CO}=\frac{{ k}_{{H}_{2}}.{b}_{{H}_{2}}.{P}_{{H}_{2}}}{{\left(1+2{\left({b}_{CO}.{P}_{CO}\right)}^\frac{1}{2}.{\left({b}_{{H}_{2}}.{P}_{{H}_{2}}\right)}^\frac{1}{2} \right)}^{2}}$
FT-IV (1)	${-r}_{CO}=\frac{{k. P}_{CO}.{b}_{{H}_{2}}.{P}_{{H}_{2}}}{{\left(1+{{(b}_{{H}_{2}}.{P}_{{H}_{2}})}^\frac{1}{2}\right)}^{4}}$	FT-XII (3)	${-r}_{CO}=\frac{k.{b}_{CO}.{P}_{CO}.{{(b}_{{H}_{2}}.{P}_{{H}_{2}})}^{2}}{{\left(1+2{\left({b}_{CO}.{P}_{CO}\right)}^\frac{1}{2}.{\left({b}_{{H}_{2}}.{P}_{{H}_{2}}\right)}^\frac{1}{2} \right)}^{6}}$
FT-IV (2)	${-r}_{CO}=\frac{{k. P}_{CO}.{{(b}_{{H}_{2}}.{P}_{{H}_{2}})}^{2}}{{(1+{{(b}_{{H}_{2}}.{P}_{{H}_{2}})}^\frac{1}{2})}^{4}}$	FT-XIII	${-r}_{CO}=\frac{k.{b}_{CO}.{P}_{CO}.{b}_{{H}_{2}}.{P}_{{H}_{2}}}{{(1+2{\left({b}_{CO}.{P}_{CO}\right)}^\frac{1}{2}.{({b}_{{H}_{2}}.{P}_{{H}_{2}})}^{1/2} )}^{4}}$
FT-V	${-r}_{CO}=\frac{{k. P}_{CO}.{b}_{{H}_{2}}.{P}_{{H}_{2}}}{{\left(1+{{(b}_{{H}_{2}}.{P}_{{H}_{2}})}^\frac{1}{2}\right)}^{2}}$	FT-XIV (1)	${-r}_{CO}=\frac{{k}_{CO}.{b}_{CO}.{P}_{CO}}{{\left(1+{2\left({b}_{CO}.{P}_{CO}\right)}^\frac{1}{2}+{b}_{{H}_{2}}.{P}_{{H}_{2}}\right)}^{2}}$
FT-VI	${-r}_{CO}=\frac{{k.P}_{CO}.{{(b}_{{H}_{2}}.{P}_{{H}_{2}})}^{1/2}}{1+{{(b}_{{H}_{2}}.{P}_{{H}_{2}}) }^{1/2}}$	FT-XIV (2)	${-r}_{CO}=\frac{{ k}_{{H}_{2}}.{b}_{{H}_{2}}.{P}_{{H}_{2}}}{{\left(1+{2\left({b}_{CO}.{P}_{CO}\right)}^\frac{1}{2}+{b}_{{H}_{2}}.{P}_{{H}_{2}}\right)}^{2}}$
FT-VII (1)	${-r}_{CO}=\frac{{ k}_{CO}.{b}_{CO}.{P}_{CO}}{{\left(1+{b}_{CO}.{P}_{CO}+{{(b}_{{H}_{2}}.{P}_{{H}_{2}})}^\frac{1}{2}\right)}}$	FT-XIV (3)	${-r}_{CO}=\frac{k.{b}_{CO}.{P}_{CO}.{b}_{{H}_{2}}.{P}_{{H}_{2}}}{{\left(1+{2\left({b}_{CO}.{P}_{CO}\right)}^\frac{1}{2}+{b}_{{H}_{2}}.{P}_{{H}_{2}}\right)}^{3}}$
FT-VII (2)	${-r}_{CO}=\frac{{ k}_{{H}_{2}}.{b}_{{H}_{2}}.{P}_{{H}_{2}}}{{\left(1+{b}_{CO}.{P}_{CO}+{{(b}_{{H}_{2}}.{P}_{{H}_{2}})}^\frac{1}{2}\right)}^{2}}$	FT-XV (1)	${-r}_{CO}=\frac{k.{({b}_{CO}.{P}_{CO})}^{1/2}.{P}_{{H}_{2}}}{{\left(1+{2\left({b}_{CO}.{P}_{CO}\right)}^\frac{1}{2}\right)}^{2}}$
FT-VII (3)	${-r}_{CO}=\frac{k.{b}_{CO}.{P}_{CO}.{{(b}_{{H}_{2}}.{P}_{{H}_{2}})}^{1/2}}{{\left(1+{b}_{CO}.{P}_{CO}+{{(b}_{{H}_{2}}.{P}_{{H}_{2}})}^\frac{1}{2}\right)}^{ 2}}$	FT-XV (2)	${-r}_{CO}=\frac{k.{({b}_{CO}.{P}_{CO})}^{1/2}.{P}_{{H}_{2}}}{1+{2\left({b}_{CO}.{P}_{CO}\right)}^{1/2}}$
FT-VIII	${-r}_{CO}=\frac{k.{b}_{CO}.{P}_{CO}.{{(b}_{{H}_{2}}.{P}_{{H}_{2}})}^{2}}{{\left(1+{b}_{CO}.{P}_{CO}+{b}_{{H}_{2}}.{P}_{{H}_{2}}\right)}^{2}}$	FT-XVI	${-r}_{CO}=\frac{k.{{(b}_{CO}.{P}_{CO})}^{1/2}.{({b}_{{H}_{2}}.{P}_{CO})}^{1/2}}{{\left(1+2{\left({b}_{CO}.{P}_{CO}\right)}^\frac{1}{2}+{\left({b}_{{H}_{2}}.{P}_{{H}_{2}}\right)}^\frac{1}{2} \right)}^{2}}$
FT-IX (1)	${-r}_{CO}=\frac{{k}_{CO}.{b}_{CO}.{P}_{CO}}{1+{b}_{CO}.{P}_{CO}+{b}_{{H}_{2}}.{P}_{{H}_{2}}}$	FT-XVII	${-r}_{CO}=\frac{k.{{(b}_{CO}.{P}_{CO})}^{1/2}.{b}_{{H}_{2}}.{P}_{CO}}{{\left(1+2{\left({b}_{CO}.{P}_{CO}\right)}^\frac{1}{2}+{b}_{{H}_{2}}.{P}_{{H}_{2}} \right)}^{2}}$
FT-IX (2)	${-r}_{CO}=\frac{{ k}_{{H}_{2}}.{b}_{{H}_{2}}.{P}_{{H}_{2}}}{1+{b}_{CO}.{P}_{CO}+{b}_{{H}_{2}}.{P}_{{H}_{2}}}$	FT-XVIII	${-r}_{CO}=\frac{k.{{(b}_{CO}.{P}_{CO})}^{1/2}.{b}_{{H}_{2}}.{P}_{CO}}{{\left(1+2{\left({b}_{CO}.{P}_{CO}\right)}^\frac{1}{2}+{\left({b}_{{H}_{2}}.{P}_{{H}_{2}}\right)}^\frac{1}{2} \right)}^{3}}$
FT-IX (3)	${-r}_{CO}=\frac{k.{b}_{CO}.{P}_{CO}.{b}_{{H}_{2}}.{P}_{{H}_{2}}}{{\left(1+{b}_{CO}.{P}_{CO}+{b}_{{H}_{2}}.{P}_{{H}_{2}}\right)}^{2}}$		

The most appropriate reaction rate (R^2^) should be close to 1. Moreover, R_msd_ and MARR might have a minimum value. The statistical parameters were determined as follows:

1. Square of the coefficient of correlation function (R^2^):

7 $$\sigma =\frac{1}{{N}_{exp}}\left(\sum_{i,CO}^{{N}_{exp}}{r}_{i,CO}^{exp}\right)$$

8 $${R}^{2}=1-\frac{\sum_{i=1}^{{N}_{exp}}({{r}_{i,CO}^{exp}-{r}_{i,CO}^{cal})}^{2}}{\sum_{i=1}^{{N}_{exp}}{({r}_{i,CO}^{exp}-\sigma )}^{2}}$$

2. Root Mean Square Deviation (R_msd_)

9 $${R}_{msd}=\frac{1}{N}{\left(\sum_{i=1}^{N}({{r}_{i,CO}^{exp}-{r}_{i,CO}^{cal})}^{2}\right)}^{2}$$

3. Mean Absolute Relative Residual (MARR):

10 $$MARR=\sum_{i=1}^{{N}_{exp}}\left|\frac{{r}_{exp}-{r}_{cal}}{{r}_{exp}}\right|*\frac{100}{{N}_{exp}}$$

Comparing experimental data to the model equation demonstrates that the FT-VII (3) model is in good agreement with the empirical results. In this scenario, carbon monoxide and hydrogen atoms are reacted while adsorbed on the surface of a catalyst.

11 $${CO}^{*}+{H}^{*}\stackrel{{k}_{p}}{\to }{CHO}^{*}+{H}^{*}\stackrel{{k}_{p1}}{\to }{CHOH}^{*}$$

12 $${CHOH}^{*}+{H}^{*}\stackrel{{k}_{p2}}{\to }{CH}_{2}{OH}^{*}+{H}^{*}\stackrel{{k}_{p3}}{\to }{CH}_{3}{OH}^{*}$$

13 $${CH}_{3}{OH}^{*}\stackrel{{k}_{p4}}{\to }{{CH}_{2}}^{*}+{H}_{2}O$$

All the reactions are series and have the same reaction rate. If the surface reaction controls the reaction rate, then the CO consumption rate is determined as below:

14 $$-{r}_{CO}={k}_{p}{\theta }_{CO}{\theta }_{H}$$

and for H_2_ surface adsorption:

15 $${H}_{2}+2S\stackrel{{K}_{ads,{H}_{2}}}{\leftrightarrow }{2H}^{*}$$

16 $${k}_{ads,{H}_{2}}{P}_{{H}_{2}}{C}_{V}^{2}-{k}_{des,{H}_{2}}{\theta }_{H}^{2}=0$$

17 $${b}_{{H}_{2}}=\frac{{k}_{ads,{H}_{2}}}{{k}_{des,{H}_{2}}}$$

where b_H2_ is equilibrium constant of H_2_ adsorption step

18 $${\theta }_{H}={({b}_{{H}_{2}}{P}_{{H}_{2}})}^{0.5}{C}_{V}$$

19 $${\theta }_{CO}={b}_{CO}{P}_{CO}{C}_{V}$$

20 $${C}_{V}=1-\sum \theta =1-\left({\theta }_{CO}+{\theta }_{H}\right)$$

21 $${C}_{V}=1-\left({b}_{CO}{P}_{CO}+{\left({b}_{{H}_{2}}{P}_{{H}_{2}}\right)}^{0.5}\right){C}_{V}$$

22 $${C}_{V}=\frac{1}{1+\left({b}_{CO}{P}_{CO}+{\left({b}_{{H}_{2}}{P}_{{H}_{2}}\right)}^{0.5}\right)}$$

Finally the CO consumption rate is obtained as below:

23 $$-{r}_{CO}=\frac{{k}_{p}{b}_{CO}{P}_{CO}({{b}_{{H}_{2}}{P}_{{H}_{2}})}^{0.5}}{{\left(1+{b}_{CO}{P}_{CO}+{\left({b}_{{H}_{2}}{P}_{{H}_{2}}\right)}^{0.5}\right)}^{2}}$$

The constant coefficient and validation parameters are estimated and presented in Table 6. The MARR percentage of the FT-VII(3) model is 9.65. The model, however, shows less deviation from the experimental data and is consistent with it. The MARR% values of the other obtained kinetic models are presented in Table 7; as it was shown, the FT-VII(3) model having the minimal MARR value fits the experimental data well. The best-fitted kinetic model is an enolic mechanism; in this mechanism, the base component (CHOH) is formed by partial hydrogenation of the absorbed carbon monoxide. According to previous research^42,43^, the enolic mechanism is much better than the carbide mechanism for bimetallic oxide catalysts.

**Table 6:** Values of kinetic parameters of FT-VII(3) model.

Parameters	Value
K_0_ (mol/min.g_cat._)	0.01756
E_a_ (kj/mol)	106.25
b_CO_ (1/bar)	0.4994
b_H2_ (1/bar)	0.1203
R^2^	0.9728
MARR (%)	9.65

**Table 7:** Parameters and mean absolute relative residuals (MARR) for the FT kinetic models.

Kinetic model	k /b_CO /_ b_H2_	MARR%	Kinetic model	k /b_CO /_ b_H2_	MARR%
FT-I (1)	1.47E − 1/14.84E0/1.47E − 1	42	FT-X (1)	9.05E − 3/49.88E0/–	10.8
FT-I (2)	2.16E − 3/14.84E0/–	10.3	FT-X (2)	9.92E − 2/2.57E − 5/–	53.7
FT-II (1)	2.64E − 3/–/5.21E − 1	14.3	FT-XI	3.97E − 3/2.91E − 2/–	34.3
FT-II (2)	2.49E − 4/1010.70E0	32.6	FT-XII (1)	5.46E − 1/2.59E − 3/1.87E0	20.3
FT-III	1.44E − 4/–/101.96E0	59.3	FT-XII (2)	7.75E − 1/9.47E-5/2.7E0	10.69
FT-IV (1)	9.63E − 3/–/1.11E0	29.4	FT-XII (3)	2.15E0/1.49E − 1/2.89E − 2	10.49
FT-IV (2)	1.42E − 3/–/4.67E − 1	37.2	FT-XIII	1.43E − 1/6.63E − 2/6.64E − 2	11.46
FT-V	4.94E − 4/–/1.50E0	32.9	FT-XIV (1)	9.15E − 3/1.50E0/2.61E − 1	54.7
FT-VI	5.46E − 2/–/3.33E − 6	34.8	FT-XIV (2)	1.33E − 2/8.11E − 2/5.88E − 3	13.1
FT-VII (1)	3.12E − 3/3.04E0/1.10E0	18.6	FT-XIV (3)	6.68E − 2/1.69E0/101.9E0	10.3
FT-VII (2)	5.64E − 3/5.56E − 1/6.23E − 2	18.3	FT-XV (1)	2.38E − 2/102.0E0/–	13.0
FT-VII (3)	1.86E0/4.99E − 1/1.20E − 1	9.65	FT-XV (2)	2.31E0/1.36E − 9/–	33.5
FT-VIII	1.18E − 4/9.06E0/8.24E0	22.1	FT-XVI	2.77E − 1/5.10E − 1/9.86E − 4	12.81
FT-IX (1)	1.99E − 3/1.06E0/1.70E0	13.0	FT-XVII	25.0E0/2.50E − 4/2.01E0	18.9
FT-IX (2)	3.11E − 3/5.80E − 1/2.06E − 1	12.4	FT-XVIII	72.66E0/7.16E − 4/2.01E0	9.96
FT-IX (3)	9.79E − 1/5.87E − 2/3.63E − 3	29.4			

**Figure 9:**
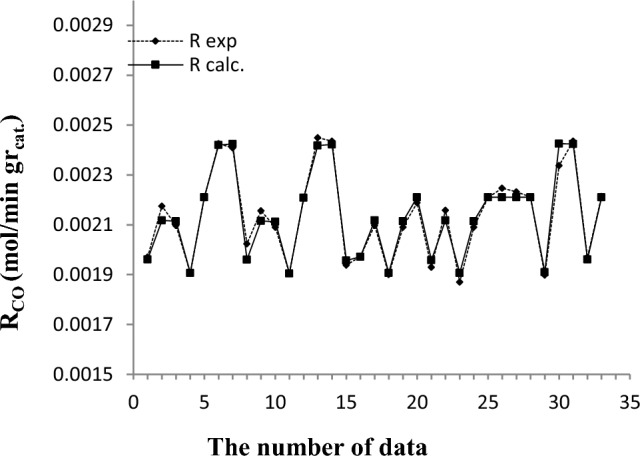
The difference between experimental and calculated reaction rate.

In addition, Fig. 9 depicts the comparison between experimental data and the calculated CO consumption rate. Polymath software shows that the experimental and calculated rates were close together and at some point, the experimental and calculated data were overlapped.

**Figure 10:**
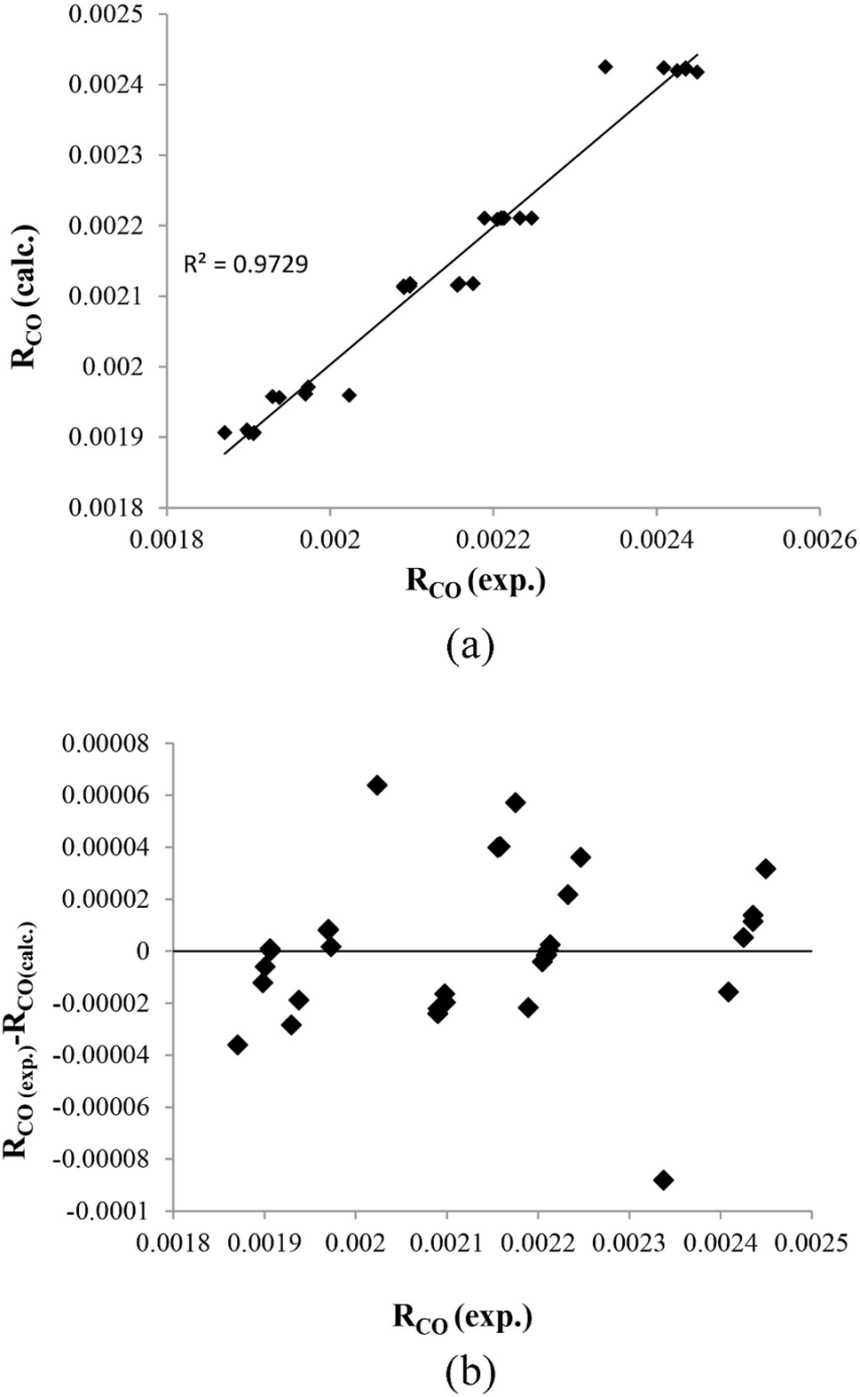
The calculated vs. the experimental reaction rate (**a**) normal plot, (**b**) residual plot.

The reaction rate is determined using the model equation and compared with the empirical data (Fig. 10a and b). The compatibility between the model and the experimental data is demonstrated by the closeness of the data to the straight line and the symmetry shape around the straight line.

In addition, Fig. 10b presents the calculated against the experimental reaction rate, and it shows that the calculated reaction rate is acceptable for predicting Fischer–Tropsch synthesis. The activation energy of a reaction is obtained by fitting data with the Arrhenius equation in various conditions. The activation energy of hydrocarbon formation is mostly between 75 to 110 kJ/mol^37–39^. In the current study, the activation energy value is 106.25 kJ/mol. which is close to activation energy reported previously 106.2 kJ/mol by abdollahi et al. and 100and 103 kJ/mol yange et al. and storch et al.^40–42^. Nevertheless, it was substantially higher than the value of 80.63 and 66.01 kJ/mol which are reported by davies et al.^43^ and mansouri et al.^16^. Although the activation energy shows the importance of the diffusion interface, the high activation energies indicate the absence of diffusion effects in the Fischer–Tropsch reaction. Therefore, the pore diffusion restriction led to the low activation energy in the Fischer–Tropsch reaction. The pore size and catalyst structure correspond with the catalyst's preparation method and component^16,44^.

## Conclusion

The performance of LaFe_0.7_Co_0.3_O_3_ perovskite catalyst and kinetic of Fischer–Tropsch synthesis (CO conversion) were investigated in a fixed bed reactor under various operating conditions (e.g. H_2_/CO: 1–2, pressure: 10–20 barg, temperature: 240–300 °C, and GHSV: 3000 1/h). Several Langmuir–Hinshelwood–Hougen–Watson (LHHW) rate equations were derived. The unknown kinetic parameters such as R^2^, R_msd_, and MARR were estimated using empirical data in Polymath software. In addition, the kinetic parameters were estimated with non-linear regression and the results show that the FT-VII model predicts CO consumption with high compatibility. Finally, the activation energy was determined with respect to the Arrhenius equation and the optimum value of 106.25 kJ/mol was estimated under various operating conditions. The kinetic parameters correspond with the preparation method and catalyst component. Therefore, the perovskite catalyst is activated at a higher temperature, and consequently, the coking issue is diminished during the operation.

## Data Availability

All experimental data were published in the current article. The additional data and information will be provided to individuals upon official request to the corresponding author [Seyed Hasan Hashemabadi].

## List of symbols

*a*: Adsorption parameter
*b*: Adsorption parameter
*C*_*V*_: Free active sites
*d*: Mean size of crystal
*E*: Activation energy (kJ/mol)
*F*_*CO*_: Molar flow rate of CO at the inlet (mol min^−1^)
*K*: Dimensionless shape factor
*k*: Reaction rate constant
*N*: Number experimental data points
*P*_*CO*_: CO pressure (bar)
*P*_*H2*_: Hydrogen pressure (bar)
*-r*_*co*_: The consumption rate of CO (mol/g_cat_ min)
*-R*_*FT*_: Rate of reaction (mol/ g_cat_ min)
*W*: Catalyst mass (gr)
*x*: Conversion
*β*: Line broadening at half the maximum intensity (FWHM)
*λ*: Is the X-ray wavelength

## Superscripts

*exp*: Experimental value
*cal*: Predicted value
